# Colonization of *Fusobacterium nucleatum* is an independent predictor of poor prognosis in gastric cancer patients with venous thromboembolism: a retrospective cohort study

**DOI:** 10.1186/s12959-022-00447-2

**Published:** 2023-01-04

**Authors:** Chang Liu, Zhou Yang, Xiance Tang, Fangfang Zhao, Mengke He, Changpeng Liu, Dongmin Zhou, Lifeng Wang, Bo Gu, Yiqiang Yuan, Xiaobing Chen

**Affiliations:** 1 The Second School of Clinical Medicine, Southern Medical University, Guangzhou, Guangdong 510280 China; 2 Department of Critical Care Medicine, The Affiliated Cancer Hospital of Zhengzhou University & Henan Cancer Hospital, Zhengzhou, Henan 450008 China; 3 Department of Biostatistics, State Key Laboratory of Organ Failure Research, Guangdong Provincial Key Laboratory of Tropical Disease Research, School of Public Health, Southern Medical University, Guangzhou, Guangdong 510515 China; 4 Department of Medical Records, Office for DRGs, The Affiliated Cancer Hospital of Zhengzhou University & Henan Cancer Hospital, Zhengzhou, Henan 450008 China; 5 Department of Gastrointestinal Oncology, The Affiliated Cancer Hospital of Zhengzhou University & Henan Cancer Hospital, No.127 Dongming Road, Zhengzhou, Henan 450008 China; 6 Department of Medical Imaging, The Affiliated Cancer Hospital of Zhengzhou University & Henan Cancer Hospital, Zhengzhou, Henan 450008 China; 7 Department of Ultrasound Therapy, The Affiliated Cancer Hospital of Zhengzhou University & Henan Cancer Hospital, Zhengzhou, Henan 450008 China; 8 Department of Cardiovascular Medicine, The 7th People’s Hospital of Zhengzhou, Henan Cardiovascular Hospital Affiliated to Southern Medical University/The Second School of Clinical Medicine, Southern Medical University, No.17 Jingnanwu Road, Zhengzhou, Henan 450016 China; 9 Department of Cardiovascular Medicine, Henan Provincial Chest Hospital, No.1 Weiwu Road, Zhengzhou, Henan 450008 China

**Keywords:** *Fusobacterium nucleatum*, Gastric cancer, Venous thromboembolism, Splanchnic vein thrombosis, Platelet-lymphocyte ratio

## Abstract

**Background:**

*Fusobacterium nucleatum* (*F. nucleatum*) often colonizes cancerous gastric tissues and is characterized by the promotion of platelet aggregation and the development of visceral thrombosis. Venous thromboembolism (VTE) leads to a significant increase in the mortality of gastric cancer (GC) patients. However, the relationship between the colonization of *F. nucleatum* and the prognosis of GC patients is still unknown.

**Aim:**

The aim of this study was to explore whether the colonization of *F. nucleatum* is related to the prognosis of GC patients complicated with VTE and to explore other potential risk factors.

**Methods:**

From 2017–2021, the data of 304 patients with new VTEs during the treatment of GC at the Affiliated Cancer Hospital of Zhengzhou University were collected. Fluorescence in situ hybridization of *F. nucleatum* was performed on pathological sections of cancer tissues from the patients. Survival analysis methods, including the Kaplan‒Meier method and Cox proportional hazard model, were performed.

**Results:**

*F. nucleatum* colonization was significantly associated with splanchnic vein thrombosis, higher platelet-lymphocyte ratio (PLR), and lower absolute lymphocyte count. In the multivariable Cox model, *F. nucleatum* colonization was found to be an independent risk factor for the prognosis of GC, with an adjusted HR of 1.77 (95% CI, 1.17 to 2.69 [*P* = 0.007]). In addition, patients with high PLR (HR: 2.65, *P* = 0.004) or VTE occurring during four cycles of chemotherapy (HR: 2.32, *P* = 0.012) exhibited shorter survival. Conversely, those experiencing VTE later (HR per month from diagnosis of GC: 0.95, *P* = 0.006) or using IVC filters (HR: 0.27, *P* = 0.011) had longer survival.

**Conclusion:**

Colonization of *F. nucleatum* in GC tissues was associated with lower absolute lymphocyte count and higher PLR in GC patients with VTE. *F. nucleatum* colonization also appeared to be associated with the development of VTE in specific sites, in particular the splanchnic vein. Colonization of *F. nucleatum* may potentially represent an independent predictor of poor prognosis in GC patients. Additional research is necessary to validate these findings.

**Supplementary Information:**

The online version contains supplementary material available at 10.1186/s12959-022-00447-2.

## Introduction

Venous thromboembolism (VTE) includes deep venous thromboembolism (DVT) and pulmonary embolism (PE), and different sites of VTE cause different clinical symptoms. Cancer is involved in the occurrence of VTE, and cancer-associated thrombosis (CAT) can significantly affect the lives of cancer patients [1]. Studies have shown that CAT is related to poor prognosis, and it has become the second leading cause of cancer patient death [2]. However, the influence of its occurrence time, location, various interventions, and related complications on the prognosis is not completely clear.

Gastric cancer (GC) is the fourth most common cancer in the world and the second leading cause of cancer death [3]. Among malignant tumours of the digestive system, the incidence of VTE in GC patients is second only to pancreatic cancer, and GC is one of the cancer types most prone to thrombosis and has a higher risk of VTE compared with other solid tumours [4]. Because of its high incidence in the Asian population and the relatively long survival time of patients, GC may be the most common cause of CAT, and the number of BC cases have gradually increased in the past 20 years [5].

*Fusobacterium nucleatum* (*F. nucleatum*) is a conditional pathogenic anaerobic bacterium existing in the gastrointestinal tract of normal people [6]. Its colonization in colorectal tissue can trigger a variety of pro-cancer mechanisms, known as oncobacterium [7]. The bacteraemia that it causes is often accompanied by thrombosis of visceral veins [8]. The reason may be that *F. nucleatum* can promote platelet aggregation by mediating endothelial cell damage [9]. Studies have shown that the bacterial flora in stomach tissue changes from *Helicobacter pylori* to *F. nucleatum* in the process of GC transformation [10]. We speculate that its colonization may be involved in the occurrence of CAT and affect the prognosis of GC patients complicated with VTE.

In summary, we designed this retrospective cohort study and uniquely added the fluorescence in situ hybridization (FISH) results of *F. nucleatum* in GC tissues to explore the influencing factors on the prognosis of GC patients complicated with VTE.

## Materials and methods

### Study population and design

The population of this study included patients who were diagnosed with GC and developed VTE complications during treatment in the Affiliated Cancer Hospital of Zhengzhou University from 2017 to 2021. The inclusion criteria were: 1) age over 18 years old; 2) histological diagnosis of gastric cancer; 3) having received antitumour treatment in our hospital for more than 4 weeks; 4) VTE occurred during the treatment and was clearly diagnosed and treated with antithrombotic therapy. Patients who had VTE or received chronic anticoagulation therapy before the diagnosis of GC were excluded.

We selected paraffin sections of the cancer tissues of the enrolled patients for FISH of *F. nucleatum* and confirmed the positive and negative results. From these patients' electronic medical records, we extracted demographic variables and treatment variables, including radiotherapy, surgery, and chemotherapy. The specific variables of cancer included presentation state, stage, histological type, and whether distant metastasis occurred at diagnosis. For the sake of comparison, we divided the stages into early, middle, and late stages, corresponding to stage I, stage II-III, and stage IV, respectively (the majority of the patients were in stage IV), the histological types into adenocarcinoma and nonadenocarcinoma, and the ECOG score (used to evaluate the patient's performance status) into 0 and ≥ 1. Given the low body weight of GC patients in Asia, we defined BMI > 25 kg/m^2^ as obesity. We also obtained clinical and biochemical data related to mortality, especially the Khorana VTE risk score (KRS), platelet-lymphocyte ratio (PLR), and neutrophil–lymphocyte ratio (NLR). According to the normal value range, biochemical indicators were defined as increased when they were above the upper limit (High) and decreased when they were below the lower limit (Low) (Table S1). According to the KRS standard, 0 is low risk of VTE, 1–2 is medium risk, and ≥ 3 is high risk (Table S2). We used the previously defined PLR > 260 and NLR > 3 as the critical values to distinguish the high and low risks of VTE. We also extracted the data of targeted drugs and PD-1 used that may affect the prognosis. Regarding the data of VTE occurrence and treatment, we selected the time of VTE occurrence (the time from diagnosis of GC to VTE occurrence as a continuous variable, and the time of VTE occurrence during chemotherapy or surgery was classified into four categories), location (including DVT, PE, splanchnic vein thrombosis (SVT) (Table S3), and catheter-related thrombosis), antithrombotic therapy (including thrombolysis, inferior vena cava (IVC) filter placement, low molecular weight heparin (LMWH), and direct oral anticoagulants (DOACs)), and whether there was bleeding during treatment. The above data were maintained and provided by our hospital's radiology department, ultrasound department and medical record room.

### Outcome measure

**Fig. 1:**
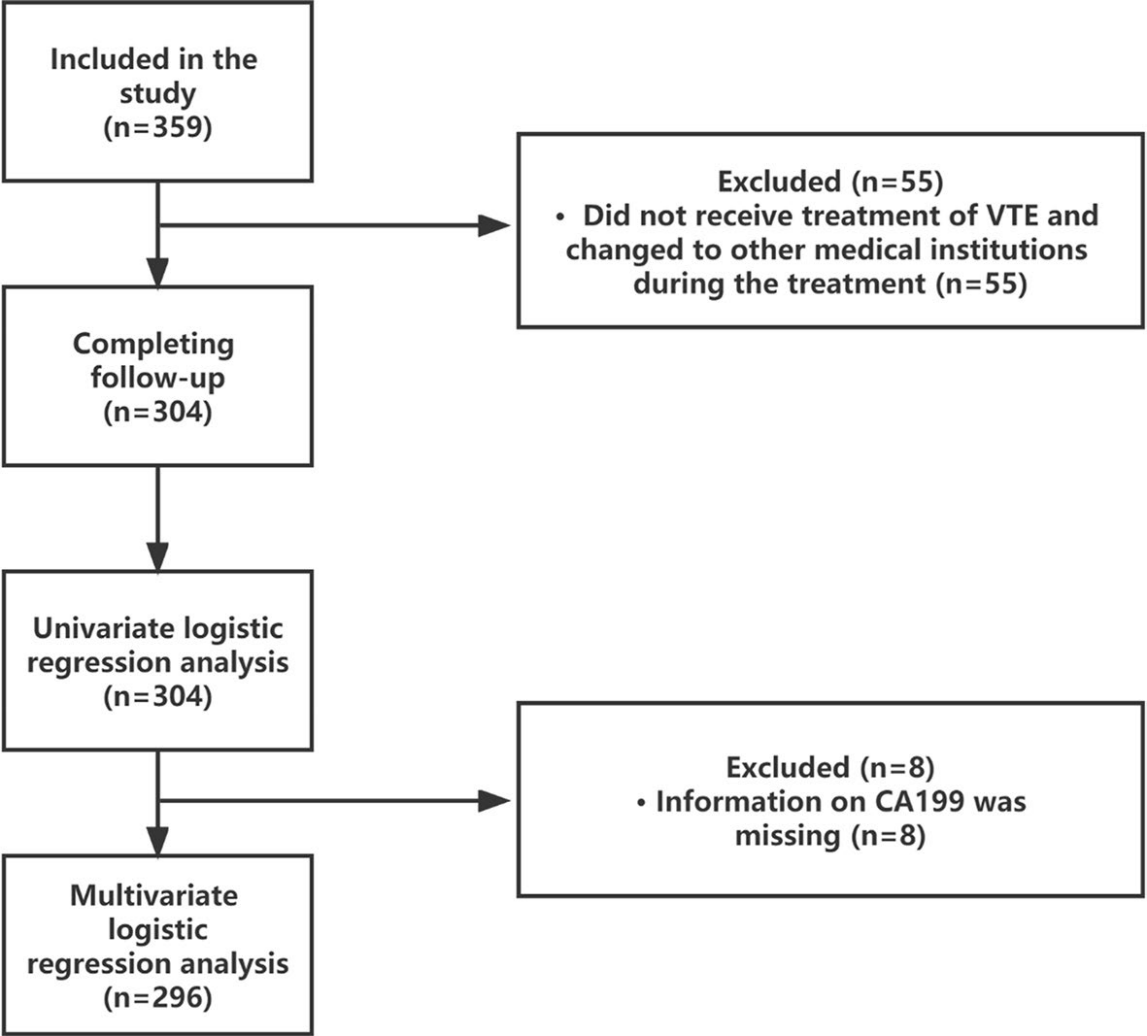
Flow diagram of selection of patients

The main outcome was overall survival (OS), that is, the survival time from the diagnosis of GC to death (or the last follow-up). VTE includes symptomatic or accidental DVTs in the upper and lower limbs, PE, SVTs, and catheter-related thromboses. All selected patients with VTE were confirmed by Doppler ultrasound, computed tomography (CT), CT angiography or ventilation perfusion scanning. From January 1, 2017, to March 31, 2021, a total of 359 eligible patients were enrolled. Among them, 304 patients completed the follow-up during the study period, while 55 patients were lost to follow-up because they did not receive treatment for VTE and changed to other medical institutions during the treatment (Fig. 1).

### Fluorescence in situ hybridization (FISH)

**Fig. 2:**
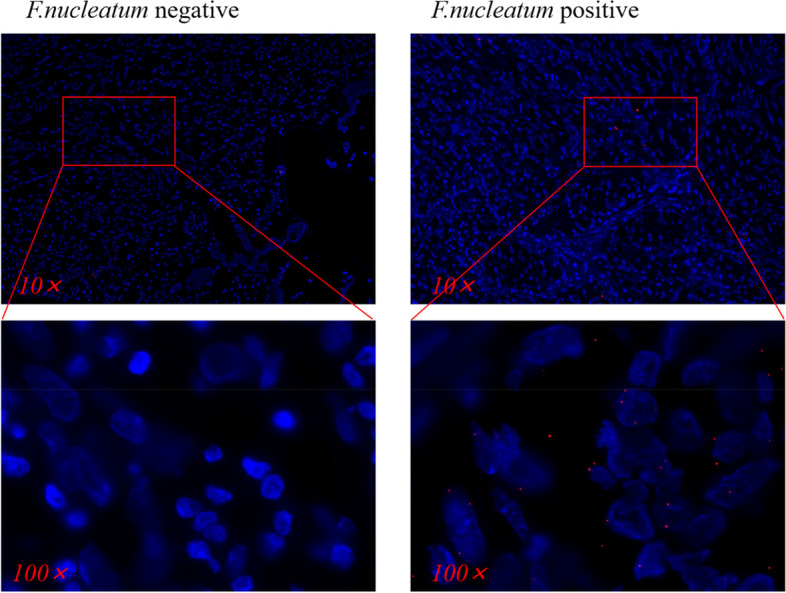
FISH results of *F. nucleatum* in paraffin sections of 304 GC patients complicated with VTE showed that 199 cases were negative and 105 cases were positive. The photos were taken at 10 × and 100 × of the objective lens

*F. nucleatum* was detected in formalin-fixed paraffin-embedded tissues by FISH. The tissue slides (4 µm) were then deparaffinized at 60 °C, followed by treatment with 100% xylene for 20 min and a graded series of ethanol at room temperature. The slides were incubated with a denatured, Cy3-labelled *F. nucleatum* probe (5 ng/μL) at 37 °C for 18 h. After five washes, the cell nuclei were stained with DAPI. Images were captured on a confocal microscope (LSM800, Zeiss), and the number of *F. nucleatum* signals was counted. Five random 100 × fields were chosen for evaluation by two pathologists blinded to tumour/normal status. The sequence for the *F. nucleatum* probe was 5’-CGCAATACAGAGTTGAGCCCTGC-3’ (Fig. 2).

### Statistical analysis

The patients included in the analyses were divided into two groups according to their FISH results of *F. nucleatum*, namely, positive and negative groups. The continuous variables are presented as the median and interquartile range (IQR), while the categorical variables are expressed as the number and percentage (%). The t test, Mann‒Whitney U test, Chi-square test, or Fisher's exact test were applied to detect the differences between the positive and negative groups when appropriate.

The Kaplan‒Meier method was employed to estimate the median survival time (MST) and survival curves, and the significant differences between survival probabilities were formally evaluated by the log-rank test. To identify the possible influencing factors on the prognosis of GC, a Cox proportional hazard model was constructed between covariates and the outcome. Specifically, univariate Cox regression was first conducted for each covariate, and those variables with a P value of less than 0.1 were considered potential prognostic factors for mortality. Furthermore, a stepwise multivariate Cox regression with a widely used backwards selection procedure was constructed on those variables to assess the adjusted risk of mortality for each factor. The final results were expressed as hazard ratios (HRs) with 95% confidence intervals (95% CIs), and factors with HRs higher than 1 increased the risk of mortality. Similarly, univariate and multivariate logistic regressions were applied to explore the potential influencing factors associated with *F. nucleatum* colonization. To avoid the possible impact of multicollinearity, which could bias the estimations of the multivariate Cox model, we additionally calculated the generalized variance inflation factor (GVIF) for each included variable. Those variables with GVIF larger than 2.24 were removed [11]. We did not impute any missing values in the original data, and *P* < 0.05 (two-sided) was considered statistically significant. All analyses were conducted in R software (Version 4.1.2).

## Results

### Patient population

A total of 304 eligible patients from 2017 to 2021 were included in the analysis, and the median follow-up time was 14.27 months (IQR: 8.04–24.58). At the end of follow-up, 169 (55.59%) patients survived, while 135 (44.41%) patients died. The overall MST of the patients in the cohort was 728 days (95% CI: 654, 895). The patient characteristics are presented in Table 1. The median age was 64 years, ranging from 24 to 87 years. The majority of the patients [224 (73.68%)] were males. The median time from diagnosis to VTE occurrence was 2.22 months (IQR: 0.96–6.45), and the most frequent type of VTE was DVT (62.83%), followed by SVT (17.76%).

**Table 1:** Summary statistics of the cohort, stratified by the FISH results of *F. nucleatum*. The continuous variables and categorical variables were respectively presented as median (IQR) and number (percentage %) among all patients or patients in each subgroup

Variables	All patients (*n* = 304)	*FISH of F. nucleatum*		*P* value
		Positive (*n* = 105)	Negative (*n* = 199)	
Age, years	64.00(14.00)	61.00(15.00)	66.00(13.00)	**0.000**
Sex, male	224(73.68%)	73(69.52%)	151(75.88%)	0.231
BMI, Obese	34(11.18%)	9(8.57%)	25(12.56%)	0.294
Smoker	122(40.13%)	41(39.05%)	81(40.70%)	0.779
HTN, Yes	75(24.67%)	31(29.52%)	44(22.11%)	0.154
DM, Yes	34(11.18%)	12(11.43%)	22(11.06%)	0.922
CVD, Yes	65(21.38%)	21(20.00%)	44(22.11%)	0.670
Histology, Adenocarcinoma	282(92.76%)	96(91.43%)	186(93.47%)	0.514
Sites of cancer				
Cardia or fundus	194(63.82%)	63(60.00%)	131(65.83%)	0.372
Corpus	45(14.80%)	14(13.33%)	31(15.58%)	-
Antrum or pylorus	51(16.78%)	23(21.90%)	28(14.07%)	-
Multiple sites	14(4.61%)	5(4.76%)	9(4.52%)	-
Stages, IV stage	101(33.22%)	47(44.76%)	54(27.14%)	**0.002**
Treatment				
Chemotherapy only	64(21.05%)	31(29.52%)	33(16.58%)	0.073
Combined surgery	218(71.71%)	67(63.81%)	151(75.88%)	-
Combined radiation	12(3.95%)	4(3.81%)	8(4.02%)	-
Combined surgery and radiation	10(3.29%)	3(2.86%)	7(3.52%)	-
CVC, Yes	140(46.05%)	46(43.81%)	94(47.24%)	0.569
Targeted drug, Yes	55(18.15%)	28(26.67%)	27(13.64%)	**0.005**
PD-1, Yes	14(4.61%)	6(5.71%)	8(4.02%)	0.568
Disease status at the time of VTE diagnosis				
During 3-wk postoperative period	54(17.76%)	9(8.57%)	45(22.61%)	**0.004**
During four cycles chemotherapy	94(30.92%)	43(40.95%)	51(25.63%)	-
During both period	14(4.61%)	5(4.76%)	9(4.52%)	-
Neither	142(46.71%)	48(45.71%)	94(47.24%)	-
Sites of VTE				
DVT	191(62.83%)	36(34.29%)	155(77.89%)	**0.000**
SVT	54(17.76%)	49(46.67%)	5(2.51%)	-
PE	22(7.24%)	11(10.48%)	11(5.53%)	-
Catheter-related thrombosis	37(12.17%)	9(8.57%)	28(14.07%)	-
Time to VTE diagnosis, months	2.22(5.49)	3.10(5.57)	1.63(4.33)	**0.033**
Antithrombotic therapy				
LMWH	155(50.99%)	55(52.38%)	100(50.25%)	0.984
DOACs	117(38.49%)	40(38.10%)	77(38.69%)	-
Thrombolysis	7(2.30%)	2(1.90%)	5(2.51%)	-
IVC filter	25(8.22%)	8(7.62%)	17(8.54%)	-
Hemorrhage, Yes	25(8.22%)	8(7.62%)	17(8.54%)	0.780
KRS, High risk	120(39.47%)	49(46.67%)	71(35.68%)	0.062
ECOG, ≥ 1	139(45.72%)	56(53.33%)	83(41.71%)	0.053
CEA, ng/ml	3.84(6.47)	3.92(6.68)	3.70(5.71)	0.328
CA199, u/ml	14.88(23.71)	15.90(28.50)	14.07(20.48)	0.117
CA724, u/ml	3.03(8.39)	5.75(15.70)	2.08(5.55)	**0.000**
Hemoglobin, g/L	109.00(22.00)	106.00(25.00)	111.00(18.50)	**0.001**
Albumin, g/L	37.50(6.98)	36.60(7.70)	37.90(6.70)	0.061
Platelet, × 10^9^/L	168.00(99.50)	184.00(98.00)	159.50(82.50)	**0.010**
Leucocyte, × 10^9^/L	5.22(3.77)	4.73(3.23)	5.35(4.09)	0.061
Neutrophil, × 10^9^/L	3.16(3.31)	3.29(2.67)	3.10(3.81)	0.850
Lymphocyte, × 10^9^/L	1.29(0.83)	1.04(0.60)	1.47(0.91)	**0.000**
Fibrinogen, g/L	2.69(1.07)	2.85(1.14)	2.62(0.99)	**0.019**
D-dimer, mg/L	1.63(3.28)	1.92(3.63)	1.52(3.02)	0.585
NLR	2.45(3.64)	3.49(3.47)	2.07(3.14)	**0.001**
PLR	123.70(112.45)	190.34(132.37)	109.65(72.91)	**0.000**

*BMI* Body Mass Index, *HTN* Hypertension, *DM* Diabetes Mellitus, *CVD* Cardiovascular Disease, *CVC* Central Venous Catheter, *VTE* Venous Thromboembolism, *DVT* Deep Venous Thromboembolism, *SVT* Splanchnic Vein Thrombosis, *PE* Pulmonary Embolism, *LMWH* Low Molecular Weight Heparin, *DOACs* Direct Oral Anticoagulants, *IVC* Inferior Vena Cava, *KRS* Khorana Risk Score, *CEA* Carcinoembryonic Antigen, *NLR* Neutrophil–Lymphocyte Ratio, *PLR* Platelet-Lymphocyte Ratio

### Association of *F. nucleatum* colonization with patient characteristics and survival

All paraffin sections of the cancer tissues of the enrolled patients were examined by *F. nucleatum* FISH to determine whether *F. nucleatum* colonization existed, among which 105 slides were positive and 199 slides were negative, and a positive result was defined as *F. nucleatum* colonization. The positive rate of *F. nucleatum* colonization was found to be significantly associated with some patient characteristics, such as age, cancer stage, site of VTE and some laboratory examinations (Table 1). After the selection of a stepwise multivariate logistic model, the VTE type of SVT (OR: 94.29; 95% CI, 27.51 to 323.12) and PE (OR: 5.45; 95% CI, 1.62 to 18.36), as well as a high level PLR (OR: 7.83; 95% CI, 2.32 to 26.40) were found to be substantially associated with positive *F. nucleatum* colonization. In addition, young age, low levels of lymphocytes, and high levels of CA724 were also significantly associated with positive *F. nucleatum* colonization (Table 2).

**Table 2:** Multivariate logistic regression analysis of variables associated with *F. nucleatum* colonization

Variables	OR	*P*
Age	0.97 (0.93, 1.00)	**0.049**
Sites of VTE		
DVT	Ref	-
SVT	94.29 (27.51, 323.12)	**0.000**
PE	5.45 (1.62, 18.36)	**0.006**
Catheter-related thrombosis	0.92 (0.29, 2.89)	0.881
KRS, High Risk vs. Median risk	2.03 (0.93, 4.41)	0.074
CA724, High vs. Normal	2.30 (1.06, 5.03)	**0.036**
Lymphocyte, Low vs. Normal	3.81 (1.74, 8.31)	**0.001**
Lymphocyte, High vs. Normal	0.00 (0.00, Inf)	0.988
PLR, High vs. Normal	7.83 (2.32, 26.40)	**0.001**

*VTE* Venous Thromboembolism, *DVT* Deep Venous Thromboembolism, *SVT* Splanchnic Vein Thrombosis, *PE* Pulmonary Embolism, *KRS* Khorana Risk Score, *PLR* Platelet-Lymphocyte Ratio

**Fig. 3:**
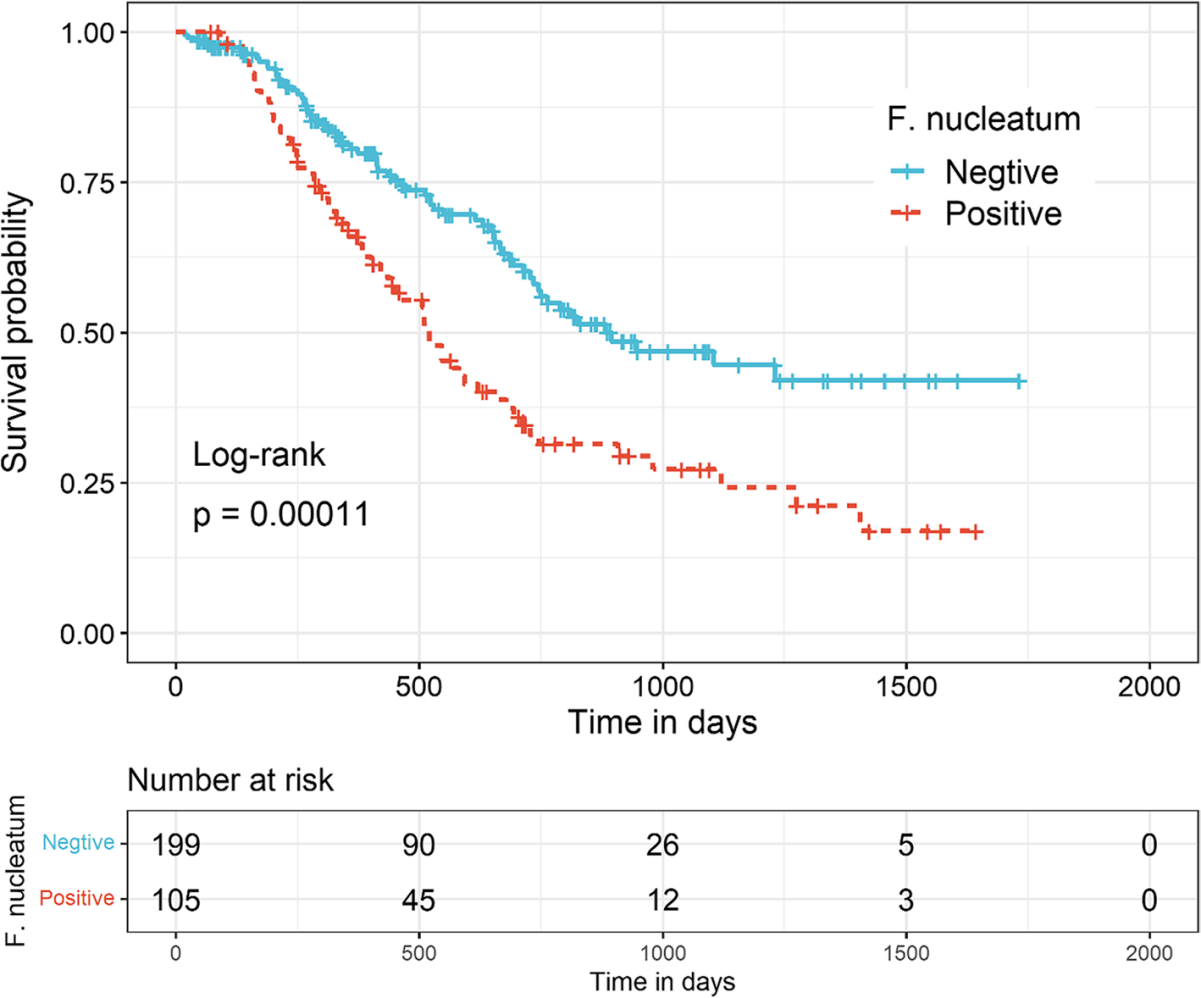
Association between overall survival and *F. nucleatum* colonization

In the Kaplan‒Meier analysis (Fig. 3), the Kaplan‒Meier curves for positive and negative *F. nucleatum* colonization were determined. Short survival was detected in patients with positive *F. nucleatum* colonization, and the MST for the positive and negative groups were 521 and 883 days, respectively, with a significant difference (log-rank test *p* < 0.001).

### Analysis of prognostic variables

The univariate analysis identified 20 eligible prognostic variables of gastric cancer for the multivariate model according to the P value threshold of 0.1, and these values included DM, sites of cancer, cancer stages, treatment, targeted drug, PD-1, disease status at the time of VTE diagnosis, sites of VTE, time to VTE diagnosis, antithrombotic therapy, KRS, CEA, CA199, CA724, platelet, lymphocyte, fibrinogen, NLR, PLR, and *F. nucleatum* (Table 2). Among them, SVT, high PLR, and positive *F. nucleatum* were associated with significantly shorter OS.

In the multivariate Cox analysis (Table 3), 296 patients were included. After adjusting for the effect of other variables, positive *F. nucleatum* colonization was still found to be an independent risk factor for the prognosis of GC, with an adjusted HR of 1.77 (95% CI, 1.17 to 2.69 [*P* = 0.007]). High PLR was also associated with significantly shorter OS (HR: 2.65; 95% CI, 1.37 to 5.12 [*P* = 0.004]). However, the VTE type was removed in the stepwise procedure. Regarding the other significant prognostic variables, DM (HR: 1.79, *P* = 0.023), multiple sites (HR: 2.89, *P* = 0.005), IV stage (HR: 2.31, *P* < 0.001), PD-1 (HR: 3.23, *P* = 0.001), VTE occurring during four cycles of chemotherapy (HR: 2.32, *P* = 0.012), high levels of CA199 (HR: 2.05, *P* < 0.001), low absolute platelet count (HR: 1.78, *P* = 0.008), and high absolute lymphocyte count (HR: 3.08, *P* = 0.020) were significantly associated with poor OS. However, late-occurring VTE (HR per month: 0.95, *P* = 0.006) and antithrombotic therapy with IVC filters (HR: 0.27, *P* = 0.011) were correlated with longer survival times.

**Table 3:** Univariate and multivariate Cox regression analysis of prognostic variables affecting mortality

**Variables**	**Univariate model**		**Multivariate model**	
	**HR**	***P***	**HR**	***P***
Age	0.99 (0.97, 1.01)	0.193		
Sex, male vs. female	0.92 (0.63, 1.36)	0.690		
BMI, Obese vs. Normal	1.10 (0.63, 1.92)	0.738		
Smoker, Yes vs. No	0.78 (0.55, 1.10)	0.151		
HTN, Yes vs. No	0.83 (0.55, 1.24)	0.361		
DM, Yes vs. No	1.71 (1.07, 2.73)	**0.025**	1.79 (1.09, 2.97)	**0.023**
CVD, Yes vs. No	1.15 (0.77, 1.71)	0.501		
Histology, Adenocarcinoma vs. Others	0.61 (0.34, 1.10)	0.102		
Sites of cancer				
Cardia or fundus	Ref	-	Ref	-
Corpus	1.47 (0.93, 2.32)	0.101	1.11 (0.67, 1.82)	0.686
Antrum or pylorus	1.28 (0.80, 2.06)	0.299	1.15 (0.69, 1.93)	0.588
Multiple sites	3.38 (1.68, 6.79)	**0.001**	2.89 (1.38, 6.02)	**0.005**
Stages, IV stage vs. Others	3.22 (2.27, 4.55)	**0.000**	2.31 (1.54, 3.46)	**0.000**
Treatment				
Chemotherapy only	Ref	-		
Combined surgery	0.36 (0.24, 0.52)	**0.000**		
Combined radiation	1.00 (0.48, 2.08)	0.998		
Combined surgery and radiation	0.27 (0.10, 0.76)	**0.013**		
CVC, Yes vs. No	1.26 (0.90, 1.77)	0.178		
Targeted drug, Yes vs. No	2.08 (1.42, 3.06)	**0.000**		
PD-1, Yes vs. No	2.51 (1.31, 4.82)	**0.006**	3.23 (1.62, 6.42)	**0.001**
Disease status at the time of VTE diagnosis				
During 3-wk operative period	Ref	-	Ref	-
During four cycles chemotherapy	2.21 (1.24, 3.93)	**0.007**	2.32 (1.21, 4.46)	**0.012**
During both period	1.69 (0.65, 4.37)	0.280	1.92 (0.71, 5.18)	0.200
Neither	1.44 (0.82, 2.52)	0.209	2.04 (1.00, 4.17)	0.050
Sites of VTE				
DVT	Ref	-		
SVT	1.96 (1.31, 2.91)	**0.001**		
PE	1.18 (0.61, 2.29)	0.620		
Catheter-related thrombosis	0.76 (0.43, 1.35)	0.352		
Time to VTE diagnosis	0.98 (0.95, 1.00)	**0.042**	0.95 (0.92, 0.99)	**0.006**
Antithrombotic therapy				
LMWH	Ref	-	Ref	-
DOACs	0.74 (0.52, 1.05)	0.095	0.93 (0.58, 1.48)	0.751
Thrombolysis	0.49 (0.15, 1.55)	0.225	0.31 (0.09, 1.06)	0.063
IVC filter	0.41 (0.17, 1.02)	0.056	0.27 (0.10, 0.75)	**0.011**
Hemorrhage, Yes vs. No	0.90 (0.44, 1.84)	0.778		
KRS, High Risk vs. Median risk	1.39 (0.99, 1.96)	0.059		
ECOG, ≥ 1 vs. 0	0.92 (0.65, 1.29)	0.621		
CEA, High vs. Normal	1.55 (1.09, 2.20)	**0.014**		
CA199, High vs. Normal	2.73 (1.91, 3.89)	**0.000**	2.05 (1.37, 3.06)	**0.000**
CA724, High vs. Normal	2.00 (1.39, 2.88)	**0.000**		
Hemoglobin, Low vs. Normal	0.96 (0.55, 1.67)	0.882		
Albumin, Low vs. Normal	1.06 (0.74, 1.51)	0.764		
Platelet, Low vs. Normal	1.47 (1.02, 2.12)	**0.038**	1.78 (1.16, 2.73)	**0.008**
Platelet, High vs. Normal	1.41 (0.65, 3.05)	0.383	0.94 (0.36, 2.44)	0.899
Leucocyte, Low vs. Normal	1.11 (0.72, 1.71)	0.624		
Leucocyte, High vs. Normal	1.17 (0.71, 1.92)	0.542		
Neutrophil, Low vs. Normal	1.19 (0.78, 1.80)	0.416		
Neutrophil, High vs. Normal	1.33 (0.85, 2.09)	0.211		
Lymphocyte, Low vs. Normal	1.77 (1.26, 2.51)	**0.001**	0.97 (0.62, 1.50)	0.877
Lymphocyte, High vs. Normal	1.50 (0.60, 3.71)	0.385	3.08 (1.19, 7.97)	**0.020**
Fibrinogen, Low vs. Normal	0.80 (0.45, 1.43)	0.459		
Fibrinogen, High vs. Normal	2.26 (1.31, 3.89)	**0.003**		
D-dimer, High vs. Normal	1.43 (0.75, 2.75)	0.277		
NLR, High vs. Normal	1.45 (1.03, 2.04)	**0.033**		
PLR, High vs. Normal	1.97 (1.21, 3.20)	**0.006**	2.65 (1.37, 5.12)	**0.004**
*F. nucleatum*, Positive vs. Negative	1.92 (1.37, 2.70)	**0.000**	1.77 (1.17, 2.69)	**0.007**

*BMI* Body Mass Index, *HTN* Hypertension, *DM* Diabetes Mellitus, *CVD* Cardiovascular Disease, *CVC* Central Venous Catheter, *VTE* Venous Thromboembolism, *DVT* Deep Venous Thromboembolism, *SVT* Splanchnic Vein Thrombosis, *PE* Pulmonary Embolism, *LMWH* Low Molecular Weight Heparin, *DOACs* Direct Oral Anticoagulants, *IVC* Inferior Vena Cava, *KRS* Khorana Risk Score, *CEA* Carcinoembryonic Antigen, *NLR* Neutrophil–Lymphocyte Ratio, *PLR* Platelet-Lymphocyte Ratio

## Discussion

*F. nucleatum* is a gram-negative anaerobe that exists in the oral cavity and gastrointestinal tract [6]. It rarely causes infection, but when it does, it is often accompanied by thrombosis of the visceral veins [12]. In recent years, research on *F. nucleatum* in colorectal cancer has brought it back as a hot research topic. Studies have confirmed that its colonization in cancer tissue is involved in biological behaviours, such as proliferation, migration and drug resistance of tumour cells [13], but there is a lack of relevant research on whether it is related to the high incidence of VTE in cancer patients, the location and prognosis. In this study, we divided patients into positive and negative *F. nucleatum* colonization groups based on the FISH results of *F. nucleatum* in paraffin sections of cancer tissues of the enrolled GC patients. In our cohort, the patients with colonization of *F. nucleatum* in cancer tissues had poor prognoses. We also found that patients with *F. nucleatum* colonization in cancer tissues had a disease course more frequently complicated by SVT and PE. In addition, colonization of *F. nucleatum* was associated with lower lymphocyte count and higher PLR in GC patients complicated with VTE. Taken together, these observations may potentially allow to speculate that colonization of *F. nucleatum* may exert pro-inflammatory and pro-thrombotic effects, thus affecting prognosis in this patient population. Additional preclinical and clinical research is however necessary to confirm this potential association, and to better characterize the underlying molecular features.

As an index to evaluate the inflammatory state of the body, PLR has been suggested to be a predictor of the prognosis of GC patients [14]. In our study, we also found that higher PLR predict a poor prognosis of GC patients. In an exploratory analysis of a retrospective cohort study [15], the authors compared patients with different types of VTE and found no significant difference in OS, but patients with DVT of the upper or lower extremity, and those with non-SVT had an unfavourable survival trend. However, only 13 GC patients with VTE were involved in the study, so the exploratory analysis was questionable in its ability to detect differences in OS. In our cohort study, we included 304 GC patients with VTE and found that patients with VTE occurred after surgery and non-SVT had a favourable survival trend.

There are multiple mechanisms by which VTE may lead to a worse survival rate of GC patients. The proposed mechanisms include: 1) the occurrence of VTE represents a stronger tumour biological response; 2) various complications secondary to the occurrence and treatment of VTE could shorten the survival time [16]. The former suggests that clinicians need to administer more aggressive tumour treatment, while the latter suggests that patients need more effective and safe antithrombotic treatment. The PLR and NLR, as potential substitutes for systemic inflammation and tumour biological response, have been confirmed in patients with gastrointestinal cancer and have good prognostic value [17, 18]. The KRS has also been indicated to have prognostic value in recent large-scale cohort studies of Asians [19]. In our cohort, the earlier occurrence of VTE (HR per month: 0.95, *P* = 0.006) and the higher PLR (HR 2.65 vs. < 260, *P* = 0.004) indicated a worse prognosis, but IVC filter placement (HR: 0.27, *P* = 0.011) after VTE occurrence reversed it. This may indicate that patients' tumour biological response is involved in the correlation between VTE and worse prognosis, and more active antitumour treatment and antithrombotic therapy should be given to improve the prognosis of GC patients.

In our cohort, we found a positive association between IVC filters use and longer survival among GC patients with VTE. The IVC filters were chosen for patients who were contraindicated for anticoagulation therapy, but it avoided the fatal complications of thrombus shedding. DM, stage IV disease, multiple GC sites, treatment with PD-1, and increased CA199 were associated with worse prognosis. As a new antitumour treatment, PD-1 is not the routine treatment of GC but is the remedial treatment of advanced cancer patients. Therefore, PD-1 is greatly affected by advanced cancer, which does not represent the real impact on prognosis.

There are some limitations in this study. Firstly, the present study was a single-centre retrospective cohort study, so findings need to be confirmed in multicentre prospective studies. Patients included in this study were all from China, so results might not apply to patients of different geographic or ethnic backgrounds. In addition, although the demographic characteristics and VTE sites were similar between patients included and retained into the study and those lost to follow-up, higher proportion of stage IV GC among the patients lost to follow-up may have caused section bias (Table S4). We therefore performed an interactive analysis and found that the effect of *F. nucleatum* on mortality was independent of cancer stage (the interaction term was insignificant) (Table S5). In addition, we could only evaluate the presence of *F. nucleatum* on GC specimens but not on the bloodstream. Moreover, to account for the generally lower body mass index (BMI) of individuals of Asian ethnicity and the substantial weight loss occurring in GC patients, obesity was defined as a BMI > 25 kg/m^2^, as previously done in other studies [19, 20]. Yet, the impact of VTE on survival in this patient population requires additional investigations as we were not able to determine the exact causes of death due to the retrospective nature of this study.

## Conclusion

Colonization of *F. nucleatum* in stomach cancerous tissues was associated with lower lymphocyte count and higher PLR among GC patients who developed VTE. *F. nucleatum* colonization was also associated with the development of VTE in specific sites (i.e., SVT and PE), and may represent an independent predictor of poor prognosis in this patient population. VTE occurring earlier after GC diagnosis, and higher PLR were also associated with shorter survival, while IVC filter used after VTE was associated with better prognosis.

## Supplementary Information

**Additional file 1: Table S1**. Normal range of biochemical indexes. **Table S2**. Khorana score risk factors: predictive model for chemotherapy-associated venous thromboembolism. **Table S3**. Summary statistics (number and proportion) of the sites of splanchic vein thrombosis for 54 analyzed patients with splanchic vein thrombosis. **Table S4**. Comparison of baseline characteristics between patients included in the analysis and patients excluded for changing to other medical institution. **Table S5**. Multivariate Cox regression analysis with interaction term between cancer stage and F. nucleatum colonization.
