# Supplementary material for: Colonization of Fusobacterium nucleatum is an independent predictor of poor prognosis in gastric cancer patients with venous thromboembolism: a retrospective cohort study
Source: Thromb J. 2023 Jan 4;21:2. doi: 10.1186/s12959-022-00447-2 (PMC9811730; doi:10.1186/s12959-022-00447-2)
Supplement: Supplementary file 1 — Additional file 1: Table S1. Normal range of biochemical indexes. Table S2. Khorana score risk factors: predictive model for chemotherapy-associated venous thromboembolism. Table S3. Summary statistics (number and proportion) of the sites of splanchic vein thrombosis for 54 analyzed patients with splanchic vein thrombosis. Table S4. Comparison of baseline characteristics between patients included in the analysis and patients excluded for changing to other medical institution. Table S5. Multivariate Cox regression analysis with interaction term between cancer stage and F. nucleatum colonization. [file 12959_2022_447_MOESM1_ESM.docx]

**Table S1** Normal range of biochemical indexes

| Biochemical indexes | Range |
| --- | --- |
| CEA, ng/ml | 0-4.7 |
| CA199, u/ml | 0-27 |
| CA724, u/ml | 0-6.9 |
| Albumin, g/L | 40-55 |
| Fibrinogen, g/L | 2-4 |
| D-dimer, mg/L | 0-0.5 |
| [Leucocyte](javascript:%20void(0)), ×10^9^/L | 3.5-9.5 |
| Neutrophil, ×10^9^/L | 1.8-6.3 |
| Lymphocyte, ×10^9^/L | 1.1-3.2 |
| Platelet, ×10^9^/L | 125-350 |
| Hemoglobin, g/L | 130-175 |

**Table S2** Khorana score risk factors: predictive model for chemotherapy-associated venous thromboembolism

| Factors | Points |
| --- | --- |
| Primary cancer site  Pancreas, stomach | 2 |
| Lung, kidney, bladder, testicle, lymphoma, gynecology | 1 |
| Platelet ≥350×10^9^/L | 1 |
| Hemoglobin <100g/L or use of red cell growth factors | 1 |
| Leukocyte >11×10^9^/L | 1 |
| BMI ≥35 kg/m^2^ | 1 |

**Table S3** Summary statistics (number and proportion) of the sites of splanchic vein thrombosis for 54 analyzed patients with splanchic vein thrombosis.

| Site | N (%) |
| --- | --- |
| portal vein | 22(40.74%) |
| Splenic vein | 10(18.52%) |
| Superior mesenteric vein | 7(12.96%) |
| inferior vena cava | 6(11.11%) |
| Renal vein | 4(7.41%) |
| superior vena cava | 3(5.56%) |
| Ovarian vein | 2(3.70%) |

**Table S4** Comparison of baseline characteristics between patients included in the analysis and patients excluded for changing to other medical institution.

| Variables | All patients (n = 359) | Patients | | P value |
| --- | --- | --- | --- | --- |
|  |  | Included (n = 304) | Excluded (n = 55) |  |
| Age, years | 64.00(14.00) | 64.00(14.00) | 64.00(14.50) | 0.234 |
| Sex, male | 262(72.98%) | 224(73.68%) | 38(69.09%) | 0.480 |
| Histology, Adenocarcinoma | 332(92.48%) | 282(92.76%) | 50(90.91%) | 0.840 |
| Stages, IV stage | 139(38.72%) | 101(33.22%) | 38(69.09%) | **0.000** |
| Sites of VTE | |  |  |  |
| DVT | 231(64.35%) | 191(62.83%) | 40(72.73%) | 0.499 |
| SVT | 61(16.99%) | 54(17.76%) | 7(12.73%) | - |
| PE | 24(6.69%) | 22(7.24%) | 2(3.64%) | - |
| Catheter-related thrombosis | 43(11.98%) | 37(12.17%) | 6(10.91%) | - |

**Table S5** Multivariate Cox regression analysis with interaction term between cancer stage and *F. nucleatum* colonization.

| **Variables** | **HR** | **P** |
| --- | --- | --- |
| DM, Yes vs. No | 1.80 (1.09, 2.97) | **0.023** |
| Sites of cancer | |  |
| Cardia or fundus | Ref | - |
| Corpus | 1.11 (0.67, 1.82) | 0.694 |
| Antrum or pylorus | 1.15 (0.69, 1.93) | 0.585 |
| Multiple sites | 2.87 (1.35, 6.09) | **0.006** |
| Stages, IV stage vs. Others | 2.27 (1.32, 3.90) | **0.003** |
| PD-1, Yes vs. No | 3.23 (1.62, 6.43) | **0.001** |
| Disease status at the time of VTE diagnosis | | |
| During 3-wk postoperative period | Ref | - |
| During four cycles chemotherapy | 2.32 (1.21, 4.47) | **0.012** |
| During both period | 1.91 (0.70, 5.17) | 0.203 |
| Neither | 2.04 (1.00, 4.18) | 0.05 |
| Time to VTE diagnosis | 0.95 (0.92, 0.99) | **0.006** |
| Antithrombotic therapy | | |
| LMWH | Ref | - |
| NOAC | 0.93 (0.58, 1.48) | 0.754 |
| Thrombolysis | 0.31 (0.09, 1.07) | 0.064 |
| IVC filter | 0.27 (0.10, 0.75) | **0.012** |
| CA199, High vs. Normal | 2.05 (1.37, 3.06) | **0.000** |
| Platelet, Low vs. Normal | 1.77 (1.15, 2.73) | **0.009** |
| Platelet, High vs. Normal | 0.94 (0.36, 2.44) | 0.895 |
| Lymphocyte, Low vs. Normal | 0.97 (0.62, 1.50) | 0.876 |
| Lymphocyte, High vs. Normal | 3.07 (1.18, 7.98) | **0.022** |
| PLR, High vs. Normal | 2.66 (1.37, 5.13) | **0.004** |
| *F. nucleatum*, Positive vs. Negative | 1.75 (1.02, 2.99) | **0.041** |
| Stages × *F. nucleatum* * | 1.03 (0.49, 2.16) | 0.934 |

* The interaction term between cancer stage and *F. nucleatum.*
